# Surface Modification of ZnO with Sn(IV)-Porphyrin for Enhanced Visible Light Photocatalytic Degradation of Amaranth Dye

**DOI:** 10.3390/molecules28186481

**Published:** 2023-09-07

**Authors:** Nirmal Kumar Shee, Hee-Joon Kim

**Affiliations:** Department of Chemistry and Bioscience, Kumoh National Institute of Technology, Gumi 39177, Republic of Korea; nirmalshee@gmail.com

**Keywords:** Sn(IV)-porphyrin, zinc oxide, adipic acid, hybrid composite, photocatalytic degradation, organic dye

## Abstract

Two hybrid composite photocatalysts, denoted as SnP/AA@ZnO and SnP@ZnO, were fabricated by a reaction of *trans*-dihydroxo[5,10,15,20-tetrakis(4-pyridyl)porphyrinato]tin(IV) (SnP) and ZnO with and without pretreatment of adipic acid (AA), respectively. In SnP@ZnO, SnP and ZnO are likely held together by a coordinative interaction between the pyridyl N atoms of SnP and the Zn atoms on the surface of ZnO. In the case of SnP/AA@ZnO, the SnP centers were robustly coupled with ZnO nanoparticles through the AA anchors. SnP/AA@ZnO exhibited largely enhanced photocatalytic activities for the degradation of anionic amaranth (AM) dye under a visible light irradiation, compared to SnP, ZnO, and SnP@ZnO. The degradation efficiency of AM by SnP/AA@ZnO was 95% within 60 min at a rate constant of 0.048 min^−1^. The remarkable photocatalytic oxidation performance of SnP/AA@ZnO was mainly attributed to the synergistic effect between SnP and ZnO. This study is valuable for the development of highly effective composite photocatalytic systems in advanced oxidation processes and is of importance for the treatment of wastewater containing dyes.

## 1. Introduction

Over the past two decades, the deterioration of water quality by discharge of wastewater containing synthetic dyes from numerous industries, such as the leather, textile, ink, cosmetic, and paper industries, has been concerning [1]. Among synthetic dyes used in the industries, more than 50% are azo dyes. The azo dyes comprise aromatic conjugated systems and are differentiated by the presence of one or more chromophoric azo bonds (−N=N−). Most of the azo dyes are water-soluble, carcinogenic, and nonbiodegradable [2,3]. The uncontrolled reductive cleavage of the azo bonds in a synthetic dye generates more toxic aromatic amines than the original dye. Owing to their peculiar properties, the removal of azo dyes from wastewater is not possible by commonly used methods such as precipitation, absorption, chemical coagulation, or biological treatments [4,5]. Recently, advanced oxidation processes (AOPs) have been widely used as a popular method for the degradation of azo dyes due to their simple operation, cost effectiveness, and high photocatalytic efficiency toward the degradation of toxic compounds to H_2_O and CO_2_ without the generation of other secondary pollution [6,7]. In AOPs, a photocatalyst absorbs light and produces reactive oxygen species (ROSs) in situ, which facilitates the degradation of toxic dyes in water [8,9].

The commonly used photocatalysts in the AOP for removal of toxic dyes are semiconducting metal oxide nanoparticles such as TiO_2_ [10], ZnO [11], SnO_2_ [12], CuO [13], MgO [14], Fe_3_O_4_ [15], V_2_O_5_ [16], and CeO_2_ [17]. Among them, TiO_2_ and ZnO have been of interest as the most practical photocatalysts due to their characteristic optical and electronic properties, nontoxicity, large surface area, low cost, and environmental friendliness. However, their substantial bandgaps (~3.37 eV for ZnO and ~3.2 eV for TiO_2_) limit the solar light to only the ultraviolet (UV) region (*λ* < 370 nm), which leads to a low photocatalytic efficiency. Moreover, a very large amount of catalyst is required during the loading process to achieve optimal degradation rates [18,19]. Various strategies, such as doping, dye sensitization, and electrodeposition, have been considered to upgrade the photocatalytic efficiency of ZnO and TiO_2_. Among them, dye sensitization is a cost-effective and well-organized method to intensify the light response range in the visible region and enhance the lifetime of photo-generated holes and electrons [20,21].

On the other hand, metalloporphyrins can absorb light in a longer range (UV-visible (Vis) light region) with high absorption coefficients. Moreover, the substantial flexibility in the molecular design, inherent aromatic electronic features, and rigid structural skeleton make them more attractive photosensitizers than other organic functional compounds. Metalloporphyrins can also amplify the photocatalytic activities of various semiconductor materials, such as TiO_2_ [22], ZnO [23], BiOBr [24], *g*-C_3_N_4_ [25], and WO_3_ [26]. After the incorporation of metalloporphyrin compounds into semiconductor materials, the resulting hybrid materials not only avoid the recombination possibility of photo-generated charge carriers but also enhance the photocatalytic efficiency via heterojunction interfaces. However, some derivatives of porphyrin do not interact with the surfaces of inorganic metal oxides due to the absence of an anchoring group. Under these circumstances, the photocatalytic activity of the hybrid photocatalysts may be reduced because the proper electron transfer does not occur [27,28,29,30]. The removal of these barriers requires a surface modification through chemical bonding between metalloporphyrin and metal oxide [31,32,33,34,35]. In addition, these chemically modified metal oxides are not only self-stabilized from hydrolysis, but also exhibit enhanced stabilities against light for further use.

**Scheme 1:**
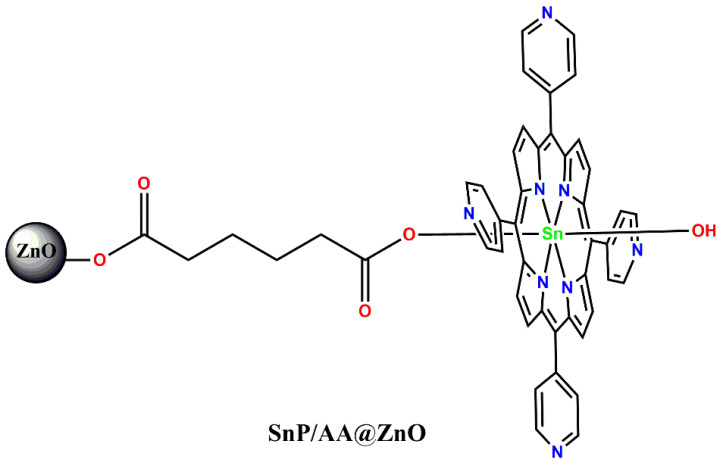
Chemical structure of the SnP/AA@ZnO hybrid composite.

Among metalloporphyrins, Sn(IV)-porphyrins have excellent coordination and photophysical features for the development of photocatalytic nanomaterials [36,37,38,39,40,41]. In particular, they can be strongly immobilized to the surface of metal oxides via the covalent-like Sn−O bond formation due to the oxophilic nature of Sn(IV)-porphyrin centers. Previously, we and others have immobilized Sn(IV)-porphyrins on the surface of TiO_2_ to fabricate hybrid nanomaterials for photocatalysis [42,43,44]. In this context, this study employs *trans*-dihydroxo[5,10,15,20-tetrakis(4-pyridyl)porphyrinato]tin(IV) (SnP) as a visible-light photosensitizer of ZnO with an anchoring group of adipic acid (AA) to fabricate a hybrid composite SnP/AA@ZnO (Scheme 1). The pyridyl-substituted metalloporphyrin SnP was considered because the N atoms of the pyridyl group can coordinate to the Zn atoms on the surface of ZnO, facilitating the electronic transfer from SnP to ZnO and improving the photocatalytic efficiency [45]. Herein, the effect of the SnP photosensitizer for the absorption of visible light, electron transfer from the excited Sn(IV)-porphyrins to the conduction band (CB) of ZnO, and enhancement in the photocatalytic activity of the hybrid nanoparticles were investigated for the degradation of the anionic amaranth (AM) dye.

The amaranth dye is widely used in the textile, medicine, food, and cosmetic industries. Owing to the improper treatment of this azo dye in industries, it may be released into the environment and cause a disastrous effect on human health. Moreover, AM is carcinogenic and mutagenic even at very low concentrations, and thus has been restricted for use in some countries. The AM dye is relatively difficult to remove using commonly used chemical, physical, or biological treatments due to its high solubility in water and toxic properties. The AM dye could be degraded only via an advanced oxidation process [46,47,48,49,50,51,52,53]. In our case, the efficiency of degradation of AM was 95% within 60 min at a rate constant of 0.048 min^−1^ by SnP/AA@ZnO, considerably higher than those of SnP, ZnO, and SnP@ZnO.

## 2. Results and Discussion

### 2.1. Fabrication and Characterization of Hybrid Photocatalysts

In a typical fabrication of the desired hybrid composite photocatalyst, a reaction mixture of ZnO nanoparticles and AA in THF was heated at reflux to form the AA-anchored ZnO. Afterward, it was mixed with SnP in THF and refluxed to fabricate the SnP/AA@ZnO composite. The SnP@ZnO composite was prepared by the reaction of SnP and pure ZnO without the AA anchor. The amount of adsorbed SnP was estimated by measuring the tin content of the hybrid composite, SnP/AA@ZnO or SnP@ZnO, by an inductively coupled plasma analysis. The loading amounts of SnP in SnP/AA@ZnO and SnP@ZnO were determined to be 0.149 and 0.103 mmol/g, respectively. The hybrid composites were characterized by various instrumental techniques.

**Figure 1:**
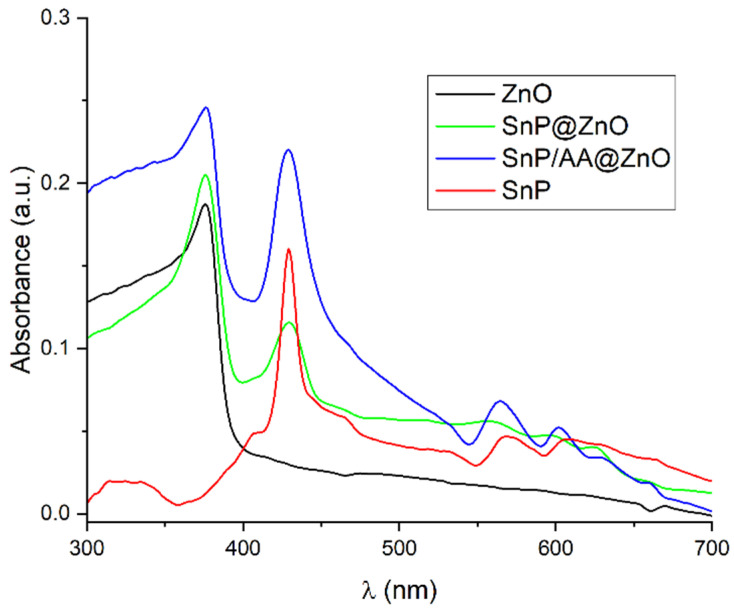
Solid-state UV-Vis absorption spectra of ZnO, SnP, SnP@ZnO, and SnP/AA@ZnO in Nujol.

Solid-state UV-Vis spectroscopy was used to characterize the light absorption properties of SnP, ZnO, SnP@ZnO, and SnP/AA@ZnO (Figure 1). SnP exhibited a strong and broad absorption band at 427 nm belonging to the Soret band, and two weak absorption bands at 566 and 606 nm corresponding to the Q bands. ZnO strongly absorbed UV light, and barely absorbed visible light. On the other hand, SnP/AA@ZnO exhibited a strong and broad light absorption band centered at 429 nm for the Soret band and three weak absorbances at 564, 602, and 627 nm for the Q bands. It also exhibited a strong UV light absorption at 376 nm due to the absorption by ZnO. This confirms the strong attachment of SnP to ZnO when anchoring groups such as AA are present on the surface of ZnO. Consequently, SnP/AA@ZnO exhibited a wider light absorption range and improved light-harvesting ability compared to SnP and ZnO. SnP@ZnO exhibited a similar absorption pattern to that of SnP/AA@ZnO, in which Soret and Q bands appeared at 428 and 558, 598, and 624 nm, respectively, along with an absorption band at 376 nm attributed to ZnO. However, the absorption bands of the SnP moiety were broader and weaker for SnP@ZnO than for SnP/AA@ZnO. These differences imply that the SnP molecules are more attached and behave more flexibly in SnP/AA@ZnO than in SnP@ZnO. The energy bandgap values were estimated through Tauc’s plot using optical absorption spectral data [54]. The determined bandgap (*E_g_*) was ~2.45 eV for SnP/AA@ZnO, which is narrower than those of SnP (~2.96 eV), ZnO (~3.24 eV), and SnP@ZnO (~2.77 eV). The enhanced light absorption and narrower bandgap of SnP/AA@ZnO can effectively improve the solar energy utilization to generate more photo-generated carriers participating in photodegradation reactions.

**Figure 2:**
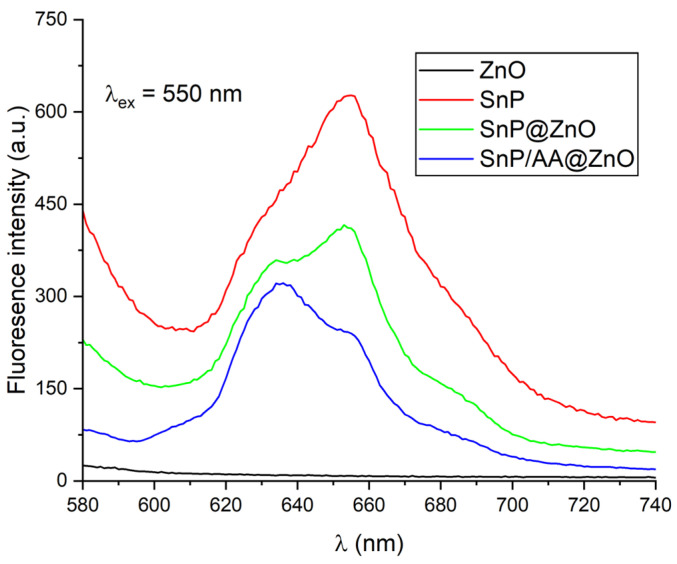
Solid-state fluorescence spectra of ZnO, SnP, SnP@ZnO, and SnP/AA@ZnO in Nujol.

Since photo-generated charge separation efficiency plays an important role in the photocatalytic process, we investigated the fluorescence properties (Figure 2). While SnP showed a strong fluorescence band centered at 655 nm, SnP@ZnO showed two bands at 634 and 653 nm. SnP/AA@ZnO also exhibited two fluorescence bands at 635 and 652 nm, but the intensity ratio was different from SnP@ZnO. SnP/AA@ZnO exhibited the lowest emission intensity, suggesting that charge separation is promoted through chemical bonding between SnP and ZnO through the anchoring group AA.

**Figure 3:**
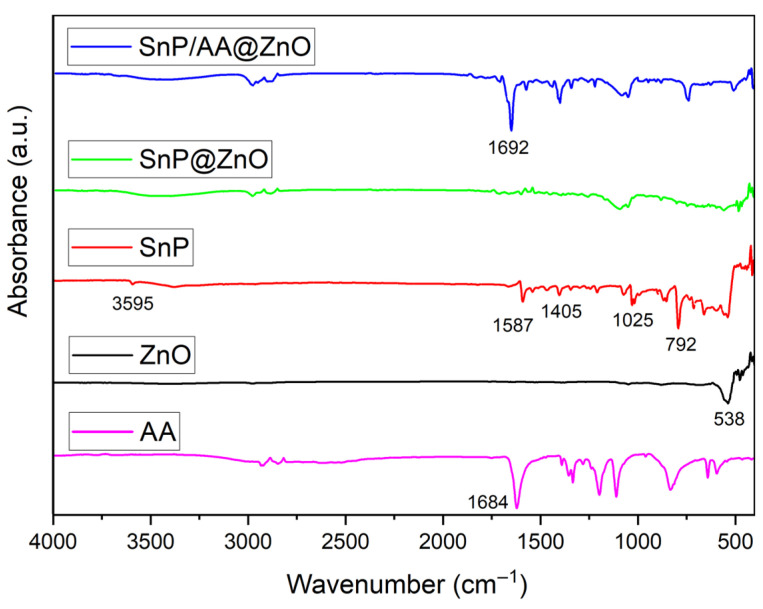
FTIR spectra of AA, ZnO, SnP, SnP@ZnO, and SnP/AA@ZnO (KBr pellets).

FTIR spectra of SnP, ZnO, SnP@ZnO, and SnP/AA@ZnO are shown in Figure 3. In the spectrum of SnP, the peaks at 1025 and 792 cm^−1^ belonged to the bending vibration of C−H and out-of-plane bending vibration of C−H in the benzene ring, respectively. The peaks at 1405 and 1587 cm^−1^ were attributed to the stretching vibrations of C−N and C=C in the pyrrole ring, respectively. The peak at 3595 cm^−1^ was assigned to the stretching vibrations of the axial OH group in SnP. For ZnO, the peak at 538 cm^−1^ was the characteristic peak of the Zn−O stretching vibration. In the spectrum of SnP@ZnO, the characteristic peak at 540 cm^−1^ was assigned to the Zn−O stretching vibration. All other peaks of either SnP or ZnO were unchanged or slightly changed. In the case of SnP/AA@ZnO, after the complexation with SnP via the AA anchoring group, the characteristic carboxylate stretching vibration band of AA appeared at 1692 cm^−1^. The shift of 8 cm^−1^ compared to the pure AA confirms the strong chemical bond between SnP and ZnO via AA. All other peaks of either SnP or ZnO were unchanged or slightly changed for SnP/AA@ZnO.

**Figure 4:**
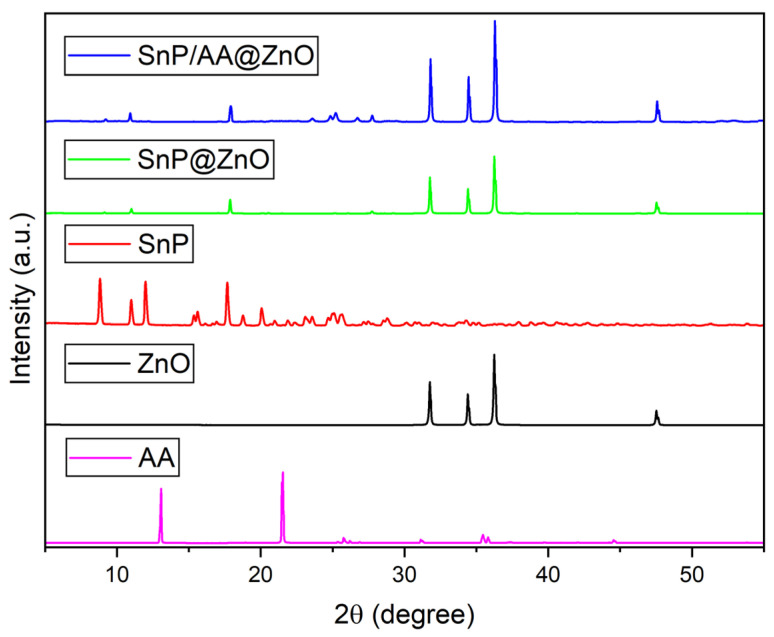
Powder XRD patterns of ZnO, SnP@ZnO, and SnP/AA@ZnO.

The crystalline structures of ZnO, SnP@ZnO, and SnP/AA@ZnO were analyzed by powder XRD, as shown in Figure 4. ZnO exhibited four major crystal peaks centered at 31.8, 34.4, 36.3, and 47.5°. In the case of SnP@ZnO, the characteristic peaks at 10.9, 17.8, and 27.7° were from SnP, while other peaks belonging to ZnO were unchanged. On the other hand, for SnP/AA@ZnO, the peaks at 22.5, 25.6, and 27.4° corresponded to AA. All other peaks were similar to those of the starting materials. Additional peak shifts to larger angles for the composites suggest a certain interaction between SnP and ZnO in either SnP@ZnO or SnP/AA@ZnO.

The TGA curves of ZnO, SnP@ZnO, and SnP/AA@ZnO are presented in Figure S1. ZnO exhibited only a very small weight loss (~1.3 wt%) between 200 and 600 °C. For the composite, the TGA loss estimates were 3.11 wt% for SnP@ZnO and 3.93 wt% for SnP/AA@ZnO between 100 and 600 °C. As shown in Figure S2, SnP@ZnO and SnP/AA@ZnO had large specific surface areas of 62.75 and 77.92 m^2^ g^−1^, respectively. In addition, type-IV adsorption–desorption isotherms were obtained, which imply a mesoporous structure. In this regard, ZnO was successfully coupled with SnP in the absence and presence of AA to form SnP@ZnO and SnP/AA@ZnO composites with high thermal stabilities, effective void space on the surface, and large surface areas.

**Figure 5:**
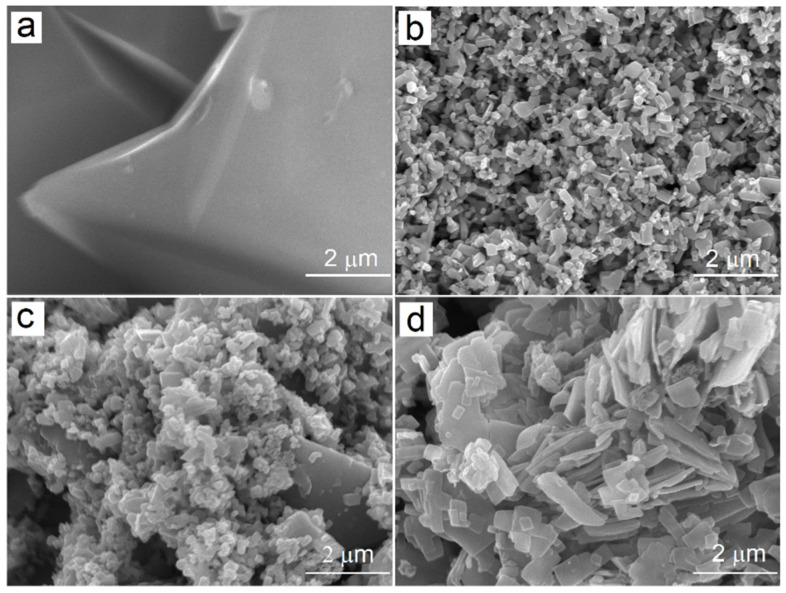
FE-SEM images of SnP (**a**), ZnO (**b**), SnP@ZnO (**c**), and SnP/AA@ZnO (**d**).

The structural and morphological development of the synthesized composites was analyzed by FE-SEM. The morphologies of SnP, ZnO, SnP@ZnO, and SnP/AA@ZnO are shown in Figure 5. The FE-SEM image in Figure 5a suggests that SnP did not exhibit any type of nanostructure. ZnO exhibited regular nanorod shapes, with an average length of 220 to 420 nm and average diameter of 40 to 120 nm (Figure 5b). For SnP@ZnO, nanorods were deposited on SnP assemblies (Figure 5c). Nanoflakes with an average length of 600−1800 nm, width of 50−300 nm, and height of ~25 nm were mainly observed in SnP/AA@ZnO (Figure 5d). The element distribution in the composite photocatalyst (SnP/AA@ZnO) was also investigated using EDS mapping, as shown in Figure S3. The results show the even distributions of Zn, O, Sn, N, and C elements in the composite, which reveals that SnP molecules were well immobilized on the ZnO nanoparticles.

**Figure 6:**
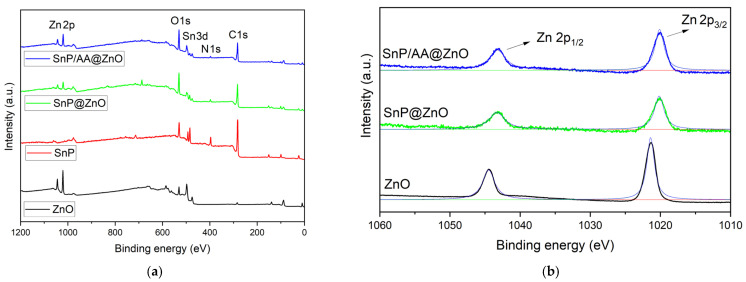
(**a**) XPS survey spectra of SnP, ZnO, SnP@ZnO, and SnP/AA@ZnO. (**b**) Deconvoluted profiles of Zn 2p core levels.

X-ray photoelectron spectroscopy (XPS) was performed to reveal the surface chemical state of SnP@ZnO and SnP/AA@ZnO. According to the survey spectra, SnP consists of Sn, C, N, and O elements, ZnO contains Zn and O elements, and SnP@ZnO and SnP/AA@ZnO contain Sn, Zn, C, N, and O elements (Figure 6a). In the Zn 2p spectrum of Figure 6b, ZnO showed two peaks at 1044.5 eV (Zn 2p_1/2_) and 1021.4 eV (Zn 2p_3/2_). For SnP@ZnO, these two peaks shifted to 1043.4 and 1020.2 eV. The corresponding peaks of SnP/AA@ZnO appeared at almost the same energies of 1043.2 and 1020.2 eV. The lower binding energy of SnP@ZnO compared to ZnO implies that the Zn 2p electron density increased due to the coordination interactions between the pyridyl N of SnP and the Zn atoms on the ZnO surface and the interfacial electron transfer between them. Deconvoluted profiles for other elements (C 1s, N 1s, O 1s, and Sn 3d) were also presented in Figure S4. In the N 1s spectrum (Figure S4b), SnP showed two peaks at 396.3 and 396.8 eV. The N 1s spectrum of SnP@ZnO can be divided into three peaks at 396.3, 397.1, and 398.6 eV. For SnP/AA@ZnO, these peaks appeared at 396.2, 396.8, and 398.4 eV. The peaks of N 1s profiles in the SnP@ZnO and SnP/AA@ZnO compared with SnP shifted to a higher binding energy, indicating a decreased N 1s electron density due to the coordination interaction between pyridyl N of SnP and the Zn atoms.

### 2.2. Photocatalytic Degradation of an Organic Dye

**Figure 7:**
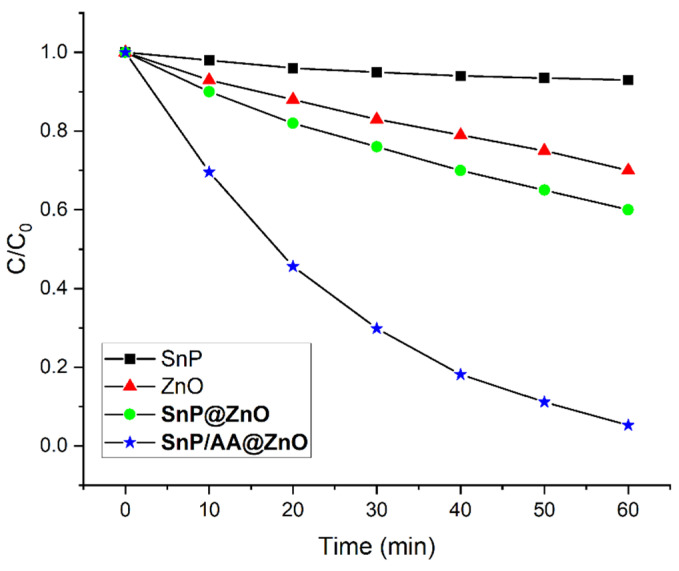
Photocatalytic degradation of AM dye in aqueous solutions (*T* = 298 K, pH = 7) under a visible light irradiation in the presence of SnP/AA@ZnO, SnP@ZnO, SnP, and ZnO.

The photocatalytic efficiencies of the composites were investigated by the degradation of the AM dye under a visible light irradiation in aqueous solutions. As shown in Figure S5, a period of approximately 25 min was required to reach the adsorption–desorption equilibrium. Approximately 3, 10, 13, and 18% of the AM dye were adsorbed by SnP, ZnO, SnP@ZnO, and SnP/AA@ZnO, respectively, which indicates that the large surface area and void spaces of ZnO can enhance the adsorption of the composites and facilitate the mass diffusion. Time-dependent absorption spectra of AM in the presence of SnP/AA@ZnO under a visible light irradiation are shown in Figure S6. A negligible decay of the AM dye was observed in the absence of visible light or the SnP/AA@ZnO photocatalyst (Figure 7).

Therefore, not only the photocatalyst but also visible light is essential for the degradation of AM dye. In Figure S6, the absorbance of the AM dye at 520 nm decreases with the increase in the visible light irradiation time. All precursors and as-prepared composite catalysts led to significant progress in the photodegradation of AM in aqueous solutions.

The degradation of AM dye in the presence of an as-prepared photocatalyst can be expressed by its degradation efficiency: (*C*_0_ − *C*)/*C*_0_, where *C* is the concentration of the AM dye at time *t* and *C*_0_ is the initial concentration at time *t*_0_. The observed degradation rate of AM was 7% for SnP, 30% for ZnO, 41% for SnP@ZnO, and 95% for SnP/AA@ZnO within 60 min of visible light irradiation (Figure 7). The SnP/AA@ZnO photocatalyst exhibited a considerably better performance for the degradation of AM dye than SnP, ZnO, and SnP@ZnO. To further interpret the reaction kinetics for the degradation of AM dye, we considered the pseudo-first-order concept, expressed by: ln(*C*_0_/*C*) = *kt*, which is widely used for photocatalytic degradation experiments if the initial concentration of the dye is low, where *k* is the pseudo-first-order rate constant for the degradation process. Based on the data in Figure 7, the reaction kinetics of the AM dye degradation are depicted in Figure S7. The first-order rate constant for the degradation of the AM dye was 0.001 min^−1^ for SnP, 0.006 min^−1^ for ZnO, 0.008 min^−1^ for SnP@ZnO, and 0.048 min^−1^ for SnP/AA@ZnO (Figure S7). The above results are encouraging compared to the reported values (Table 1) for the degradation of AM dye.

Among the photocatalysts, SnP/AA@ZnO exhibited the highest photodegradation efficiency. It could remove more than 95% of the AM dye within 60 min. To analyze the effect of SnP on the photocatalytic activity, a series of SnP/AA@ZnO were prepared with varying weight percentages of SnP to ZnO, and then the AM dye degradation rate was measured (Figure S8). The photodegradation rate of AM for the composite SnP/AA@ZnO (x = 30%) was higher than those of pure SnP and pure ZnO. As the mass ratio of SnP to ZnO increased in the composite, the rate increased and reached a maximum of 30%. The rate then slightly decreased to 40% or 50%. This suggests that the synergistic effect between SnP and ZnO is responsible for the remarkably enhanced photocatalytic activity of the SnP/AA@ZnO composite.

For practical applications, the economic efficiency depends on the recovery of the photocatalyst after degradation. In our case, the recovery of the SnP/AA@ZnO composite from the reaction mixture was very simple. After the experiment, the solid materials are filtered, washed with water, and then dried in air. The reusability of the SnP/AA@ZnO photocatalyst was important for practical application, which was demonstrated by testing recycling of SnP/AA@ZnO against AM dye degradation (Figure S9). Even after 10 consecutive cycles, SnP/AA@ZnO maintained a high efficiency for the degradation of AM dye with a reduction of only 5%, which indicates that the SnP/AA@ZnO photocatalyst exhibited a remarkable stability. In addition, the structure of SnP/AA@ZnO was analyzed after the degradation reaction to further confirm the stability of this photocatalyst. The powder XRD (Figure S10) and FE-SEM (Figure S11) results of the used SnP/AA@ZnO were similar to the initial results, which indicates that this photocatalyst was not damaged during the photocatalytic reaction.

We also aimed to optimize the reaction conditions in terms of the AM dye/photocatalyst ratio, temperature, and pH of the solution. To this end, several experiments were performed under various conditions. The degradation efficiency increased with the temperature (Figure S12). The pH of the aqueous AM dye solution affected the degradation rate of the AM dye (Figure S13). Figure S13 shows that the rate of degradation increased from pH of 3 to 7, and decreased to pH of 12. The effect of a basic pH on the rate constant was larger than that of an acidic pH. To reveal the effect of the dye/photocatalyst ratio on the degradation of the AM dye, AM solutions with various concentrations (10 to 100 mg L^−1^) were applied with certain amounts of SnP/AA@ZnO photocatalyst (50 mg every time). We observed a decrease in the degradation rate with the increase in the concentration of the AM dye (Figure S14).

A mechanism of photocatalytic degradation of the dye in SnP/AA@ZnO and SnP@ZnO systems is proposed. The possible mechanism of the photocatalytic process by SnP@ZnO is different from that of SnP/AA@ZnO. In the case of SnP@ZnO, upon irradiation of visible light, SnP and ZnO can be excited simultaneously and generate abundant electron–hole (*e*^−^/*h*^+^) pairs. Afterward, the excited electrons from SnP can easily move to the CB of ZnO through the coordination bond between the pyridyl N and Zn atoms on the surface of ZnO [56]. These excited electrons in the CB can react with O_2_ to produce superoxide radical anions (O_2_^−•^) and degrade the AM dye. ZnO holes can react with H_2_O to produce hydroxyl radicals (^•^OH) and degrade the AM dye. On the other hand, when the surface of ZnO is chemically modified with SnP via the anchoring group AA, SnP/AA@ZnO can behave as a single molecule with a lower bandgap energy compared to either SnP or ZnO. Therefore, the degradation mechanism of SnP/AA@ZnO is very similar to those of other porphyrin-based photocatalysts [40,41]. In general, the mechanism consists of five steps. In step I (Equation (1)), the photocatalyst in an aqueous solution absorbs light upon exposure to a visible light along with the AM dye. After crossing the bandgap, valence band electrons are promoted to the CB. This facilitates the production of electron–hole pairs (*e*^−^/*h*^+^) at the surface of the hybrid photocatalyst. A strong intermolecular *π*–*π* interaction among the porphyrins strengthens the electronic delocalization over the surface of the photocatalyst. This process minimizes the recombination energy of the excited electrons. These photo-generated holes (*h*^+^) react with water to form the highly reactive hydroxyl radical (^•^OH) (step II (Equation (2))). The excited electron reacts with the dissolved oxygen to produce highly reactive superoxide radical anions (O_2_^−•^) in step III (Equation (3)). These photo-generated highly reactive radicals (O_2_^−•^ and ^•^OH) react with the AM dye and degrade it into low-molecular-weight molecules, and finally, to H_2_O and CO_2_ (steps IV and V (Equations (4) and (5))).

The mechanism is summarized below for a porphyrin-based photocatalyst, P_cat_:

P_cat_ + *hν* → P_cat_* (*e*^−^ + *h*^+^), (1)

H_2_O + *h*^+^ →^•^OH + H^+^, (2)

O_2_ + *e*^−^ → O_2_^−•^, (3)

^•^OH + AM → degraded products, (4)

O_2_^−•^ + AM → degraded products. (5)

The chemical surface modification with the anchoring AA plausibly improved the surface properties of ZnO by strengthening the bonding with the SnP photosensitizers. It is likely that the bridging–anchoring groups strongly prevented the detachment of SnP from the surface of the modified ZnO during the photocatalytic process. Therefore, the strong adhesion between SnP and ZnO through AA not only increases the amount of SnP to the surface of ZnO, but also promotes the electron transfer from the excited SnP to the CB of ZnO.

The photo-generated reactive species can be detected using radical trapping reagents in the photocatalytic degradation of the AM dye [57,58]. We used *tert*-butanol (*^t^*BuOH) to capture hydroxyl radical (^•^OH), sodium azide (NaN_3_) for singlet oxygen, para-benzoquinone (*p*-BQ) for superoxide radical anion (O_2_^−•^), and ethylenediaminetetraacetic acid disodium (Na_2_-EDTA) for photo-generated holes (h^+^) during the photodegradation of AM dye in the presence of SnP/AA@ZnO. Based on the results in Figure S15, the AM dye degradation rate was critically affected in the presence of *^t^*BuOH, *p*-BQ, and Na_2_-EDTA. However, the degradation of the AM dye was not affected by the presence of NaN_3_ or singlet oxygen. Photo-generated holes (*h*^+^) are the major reactive species, compared to hydroxyl radicals (^•^OH) or superoxide radicals (O_2_^−•^), that are responsible for the catalytic degradation of AM dye in aqueous solutions.

Additionally, the photocatalytic activity of SnP/AA@ZnO in the AM degradation under various monochromatic light wavelengths was investigated (Figure S16). The variation trend in the wavelength-dependent photodegradation of the AM dye demonstrated that an optical absorption has a significant contribution toward the solar energy conversion and photocatalytic performance. SnP/AA@ZnO exhibited a small degradation ability even at *λ* > 700 nm.

**Figure 8:**
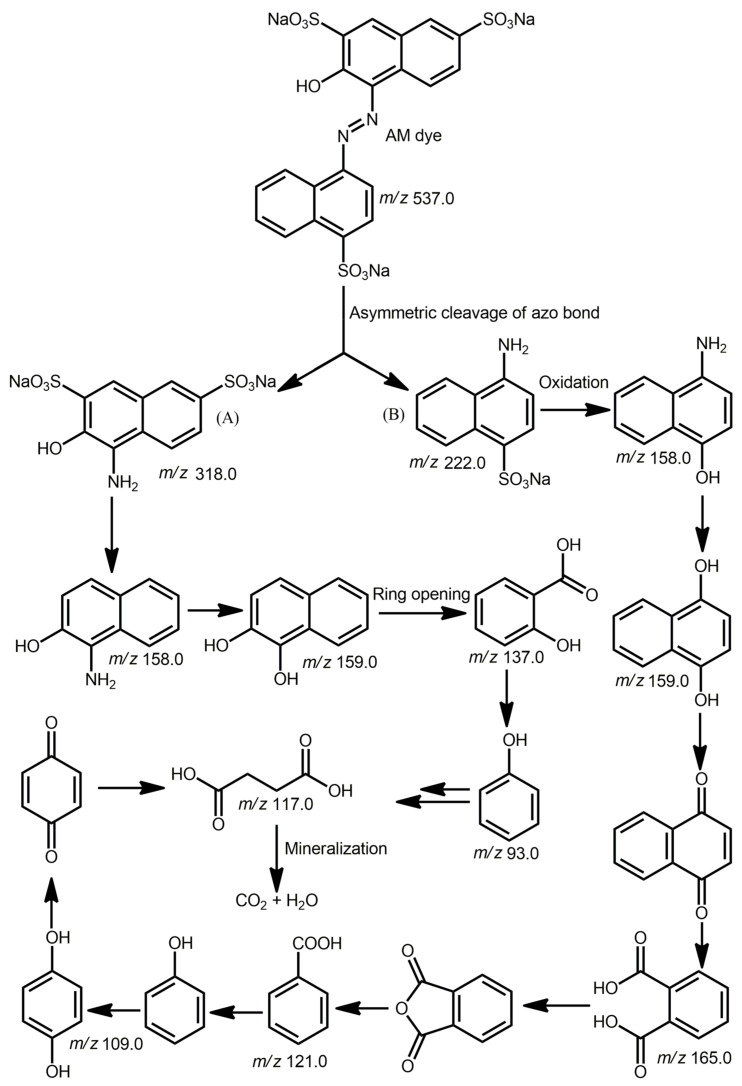
Possible intermediates upon the degradation of AM dye by the SnP/AA@ZnO photocatalyst composite.

Finally, the degraded products in the AM dye photodegradation were analyzed from the reaction mixture taken after 30 min for each photodegradation experiment by ESI mass spectrometry. New peaks in the mass spectra confirmed the degradation of AM dye to new smaller molecules [59,60]. Based on the mass spectra in Figure S17, possible intermediates for the degradation of AM dye are depicted in Figure 8. The base peak (*m*/*z* = 537.0; AM + 2H^+^ − 3Na^+^) belongs to the anionic form of the AM dye. The AM dye can undergo an asymmetric cleavage of azo bonds to form small fragments with *m*/*z* of 318.0 and 222.0. These two fragments can undergo successive aromatic ring opening and oxidation to form lower-molecular-weight species (*m*/*z* of 137.0 and 165.0). These low-molecular-weight aromatic species can then undergo successive ring cleavage and hydrolysis, leading to the formation of low-molecular-weight compounds with *m*/*z* of 117.0. Eventually, these smaller intermediates are further fragmented and mineralized into CO_2_ and H_2_O. The total organic carbon (TOC) value was calculated to estimate the removal of AM dye by the photocatalysts [61]. The TOC removal percentage was 83% for SnP/AA@ZnO and 78% for SnP@ZnO.

## 3. Materials and Methods

ZnO (particle size < 5 μm) was purchased from Sigma-Aldrich (St. Louis, MO, USA). All purchased chemicals were used without further purification unless otherwise specified. SnP was prepared according to the reported method [62]. Toluene and pyrrole were distilled from a solution containing calcium hydride. Steady-state UV-Vis spectra were acquired using a Shimadzu UV-3600 spectrophotometer (Shimadzu, Tokyo, Japan). Inductively coupled plasma optical emission spectroscopy was carried out using an iCAP™ PRO XP Duo spectrometer (Thermo Fisher Scientific Inc., Waltham, MA, USA). The Brunauer−Emmett−Teller surface area was estimated with an analyzer (BELSORP-mini volumetric adsorption equipment) using N_2_ adsorption isotherms at 77 K. Powder X-ray diffraction (XRD) patterns were acquired using a Bruker AXS D8 Advance powder X-ray diffractometer (Bruker, Billerica, MA, USA). Surface and pore size data were obtained using Autosorb-iQ and Quadrasorb SI. A thermogravimetric analysis (TGA) was carried out using an Auto-TGA Q500 instrument (TA Instruments, New Castle, DE, USA). Fourier-transform infrared (FTIR) spectroscopy (KBr) was carried out using a Shimadzu FTIR-8400S spectrophotometer (Shimadzu, Tokyo, Japan). The morphology and elemental distribution were analyzed using field-emission scanning electron microscopy (FE-SEM) (MAIA III, TESCAN, Brno, Czech Republic) with energy-dispersive X-ray spectroscopy (EDS). Electrospray ionization (ESI) mass spectra were recorded on a Thermo Finnigan Linear Ion Trap Quadrupole mass spectrometer (Thermo Fisher Scientific, MA, USA). X-ray photoelectron spectroscopy (Thermo Fisher Scientific Co., USA) with a micro-focused Al Kα source was used to analyze the chemical composition of the sample.

### 3.1. Synthesis of SnP/AA@ZnO

ZnO (1.0 g, 12.3 mmol) was added to a solution of AA (1.80 g, 12.3 mmol) dissolved in tetrahydrofuran (THF; 20 mL). The reaction mixture was refluxed for 6 h. The solid materials were filtered and washed with THF, and then dried in a vacuum for over 2 h at 70 °C. The resulting solid was then added to a solution of SnP (0.30 g, 0.39 mmol) dissolved in THF (30 mL). The reaction mixture was refluxed for 12 h. Afterward, the solid was filtered, washed with CH_2_Cl_2_, and dried in a vacuum oven for 6 h at 90 °C. SnP/AA@ZnO powder was obtained with a yield of 1.17 g.

### 3.2. Synthesis of SnP@ZnO

ZnO (1.0 g, 12.3 mmol) was added to a solution of SnP (0.30 g, 0.39 mmol) dissolved in CH_2_Cl_2_ (30 mL). Then, the reaction mixture was stirred for 12 h at room temperature. The solid was filtered, washed with CH_2_Cl_2_, and dried in a vacuum oven for 24 h at 80 °C. A SnP@ZnO powder was obtained with a yield of 1.10 g.

### 3.3. Photocatalytic Degradation

The photocatalytic efficiencies of SnP/AA@ZnO and SnP@ZnO were investigated by degradation of AM dye in aqueous solutions. The photodegradation reaction of this dye was carried out under the irradiation of a 150 W xenon arc lamp with a UV cut-off filter (ABET technologies, Old Gate Lane, Milford, CT, USA) at 298 K. In a typical procedure, 50 mg of the photocatalyst was added to a 250 mL aqueous solution of AM (50 mg L^−1^, distilled water with pH of 7) and stirred at room temperature. The reaction mixture remained in the dark for 25 min to reach adsorption–desorption equilibrium. After irradiation with visible light, 2 mL of the suspension was collected at regular intervals. The photocatalyst was collected from the solution by centrifugation, followed by filtration using a filter paper. The concentration of AM was estimated by the absorbance at 520 nm using a UV-Vis spectrophotometer.

## 4. Conclusions

ZnO was successfully modified with SnP to provide two composite photocatalysts, SnP/AA@ZnO and SnP@ZnO, by the reaction of SnP and ZnO with and without the pretreatment of AA, respectively. The SnP molecules were considerably more tightly immobilized on ZnO nanoparticles via the AA anchor in SnP/AA@ZnO compared to SnP@ZnO. In SnP@ZnO, SnP and ZnO were likely held together by a coordinative interaction between the pyridyl N atoms of SnP and the Zn atoms on the surface of ZnO. The bridging–anchoring groups strongly prohibited the separation of SnP from the surface of ZnO during the photocatalytic process. Therefore, the chemically strong attachment between SnP and ZnO through AA linkages not only increased the amount of SnP to the surface of ZnO, but also facilitated the electron transfer from the excited SnP to the CB of ZnO. This was reflected in the large enhancement in visible light photocatalytic activity for the AM dye degradation for SnP/AA@ZnO compared to SnP, ZnO, and SnP@ZnO. The largely improved photocatalytic oxidation performance of SnP/AA@ZnO compared to SnP@ZnO was mainly attributed to the synergistic effect between SnP and ZnO via the anchoring AA groups. The incorporation of SnP on the surface of ZnO not only changed the surface structure and chemical environment, but also provided a high thermodynamic stability, large porous surface area, characteristic surface morphology, and excellent photocatalytic degradation efficiency for AM dye. The high dye degradation efficiency, low catalyst loading, and high reusability make these photocatalysts more efficient than other conventional photocatalysts, such as pure TiO_2_ and pure ZnO. Accordingly, this study is valuable for the development of highly effective composite photocatalytic systems for advanced oxidation processes and is of importance for the treatment of wastewater containing dyes.

## Figures and Tables

**Table 1:** Comparison of efficiencies of photodegradation of AM by various photocatalysts.

Photocatalyst	Rate Constant (min^−1^)	Reference
W/TiO_2_	0.063	[46]
H_2_O_2_-TiO_2_	0.016	[47]
Immobilized TiO_2_	0.0098	[48]
Boron-doped diamond anode	0.046	[49]
Graphene/TiO_2_-Ag	0.0583	[50]
Anatase TiO_2_	0.042	[51]
Ag_2_O-ZnO	0.088	[52]
Photocatalytic fuel cell-electro fenton (PEC-EF)	0.007	[53]
Porphyrin nanostructure	0.031	[55]
SnP	0.001	This study
ZnO	0.006	This study
SnP@ZnO	0.008	This study
SnP/AA@ZnO	0.048	This study

## Data Availability

Not applicable.

## Supplementary Materials

The following supporting information can be downloaded at https://www.mdpi.com/article/10.3390/molecules28186481/s1. Figure S1: TGA curves of ZnO, SnP@ZnO, and SnP/AA@ZnO. Figure S2: N_2_ adsorption–desorption isotherms of SnP@ZnO and SnP/AA@ZnO. Figure S3: EDS elemental maps (C, N, O, Zn, and Sn) of SnP/AA@ZnO. Figure S4: XPS spectra of SnP, ZnO, SnP@ZnO, and SnP/AA@ZnO. Deconvoluted profiles of (a) C 1s, (b) N 1s, (c) O 1s, and (d) Sn 3d. Figure S5: AM adsorption abilities of SnP, ZnO, SnP@ZnO, and SnP/AA@ZnO. Figure S6: Photocatalytic degradation of AM dye in an aqueous solution by SnP/AA@ZnO under a visible light irradiation. Figure S7: Kinetics for the photocatalytic degradation of AM dye by SnP, ZnO, SnP@ZnO, and SnP/AA@ZnO under a visible light irradiation. Figure S8: Comparison of AM dye degradations in the presence of SnP, ZnO, and SnP/AA@ZnO with various weight percentages of SnP with respect to ZnO. Figure S9: Recyclability of SnP/AA@ZnO in the degradation of AM dye. Figure S10: Powder XRD patterns of SnP/AA@ZnO after and before the AM photodegradation. Figure S11: FE-SEM images of SnP/AA@ZnO before and after the degradation of AM dye. Figure S12: Effect of the temperature on the AM degradation in the presence of SnP/AA@ZnO. Figure S13: Effect of the pH of the solution on the degradation of AM dye in the presence of SnP/AA@ZnO. Figure S14: Effect of the initial concentration of the AM dye on the degradation with 50 mg of SnP/AA@ZnO. Figure S15: Photocatalytic degradation of AM dye in an aqueous solution by SnP/AA@ZnO with the addition of different scavengers under a visible light irradiation ([Na_2_-EDTA]_0_ = [*p*-BQ]_0_ = [NaN_3_]_0_ = [*^t^*BuOH]_0_ = 1 mM, pH = 7.0, *T* = 298 K). Figure S16: Photocatalytic activity of SnP/AA@ZnO at different wavelengths for the degradation of AM dye. Figure S17: ESI mass spectrum (negative ion mode) of the reaction mixture of AM dye with SnP/AA@ZnO after 30 min of visible light irradiation.
