# Supplementary material for: Surface Modification of ZnO with Sn(IV)-Porphyrin for Enhanced Visible Light Photocatalytic Degradation of Amaranth Dye
Source: Molecules. 2023 Sep 7;28(18):6481. doi: 10.3390/molecules28186481 (PMC10536602; doi:10.3390/molecules28186481)
Supplement: Supplementary file 1 [file molecules-28-06481-s001.zip › molecules-2576254-supplementary.pdf]

Supplementary Materials

List of contents

Figure S1. TGA curves of ZnO, SnP@ZnO, and SnP/AA@ZnO.

Figure S2. N_2 adsorption-desorption isotherms of SnP@ZnO and SnP/AA@ZnO.

Figure S3. Energy dispersive X-ray spectroscopy (EDS) elemental maps (C, N, O, Zn, and Sn) of SnP/AA@ZnO.

Figure S4. XPS spectra of SnP, ZnO, SnP@ZnO, and SnP/AA@ZnO. Deconvoluted profiles of (a) C 1s, (b) N 1s, (c) O 1s, (d) Sn 3d.

Figure S5. AM adsorption abilities of SnP, ZnO, SnP@ZnO, and SnP/AA@ZnO.

Figure S6. Photocatalytic degradation of AM dye in an aqueous solution by SnP/AA@ZnO under a visible light irradiation.

Figure S7. Kinetics for the photocatalytic degradation of AM dye by SnP, ZnO, SnP@ZnO, and SnP/AA@ZnO under a visible light irradiation.

Figure S8. Comparison of AM dye degradation in the presence of SnP, ZnO, and SnP/AA@ZnO with various weight percentages of SnP with respect to ZnO.

Figure S9. Recyclability of SnP/AA@ZnO in the degradation of AM dye.

Figure S10. Powder XRD patterns of SnP/AA@ZnO after and before the AM photodegradation.

Figure S11. FE-SEM images of SnP/AA@ZnO before and after the degradation of AM dye.

Figure S12. Effect of the temperature on the AM degradation in the presence of SnP/AA@ZnO.

Figure S13. Effect of the pH of the solution on the degradation of AM dye in the presence of SnP/AA@ZnO.

Figure S14. Effect of the initial concentration of the AM dye on the degradation with 50 mg of SnP/AA@ZnO.

Figure S15. Photocatalytic degradation of AM dye in an aqueous solution by SnP/AA@ZnO with the addition of different scavengers under a visible light irradiation ($[Na_2\text{-EDTA}]_0$ = $[p\text{-BQ}]_0$ = $[NaN_3]_0$ = $[^t\text{BuOH}]_0$ = 1mM, pH = 7.0, T = 298 K).

Figure S16. Photocatalytic activity of SnP/AA@ZnO at different wavelengths for the degradation of AM dye.

Figure S17. ESI-mass spectrum (negative ion mode) of the reaction mixture of AM dye with SnP/AA@ZnO after 30 min of visible light irradiation.

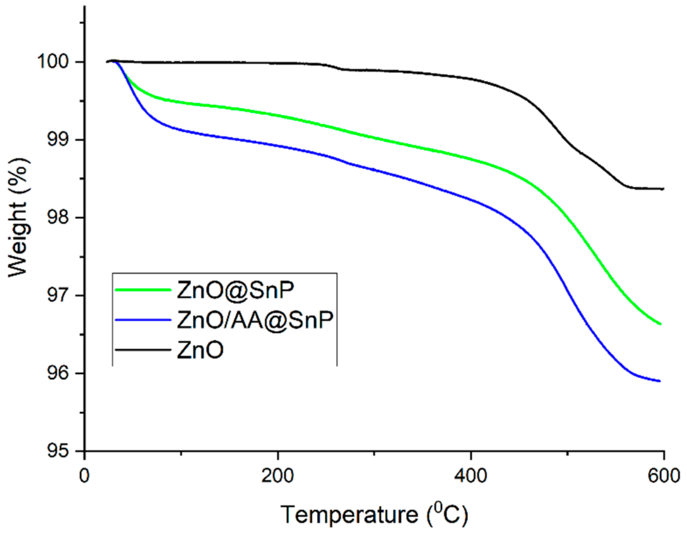

Figure S1. TGA curves of ZnO, SnP@ZnO, and SnP/AA@ZnO.

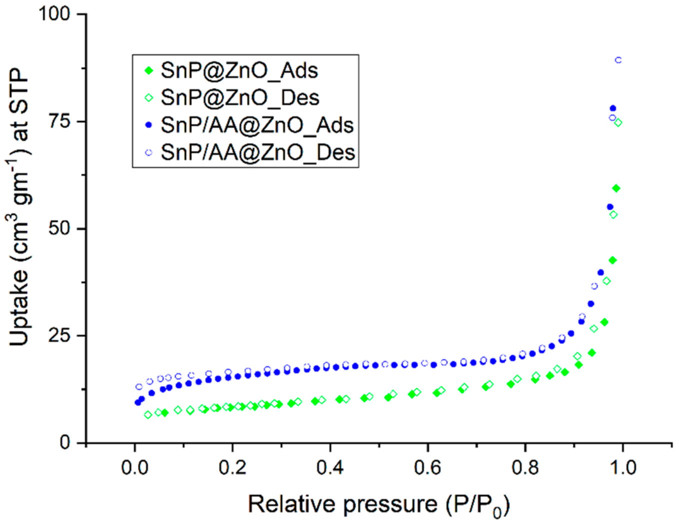

Figure S2. N_2 adsorption-desorption isotherms of SnP@ZnO and SnP/AA@ZnO.

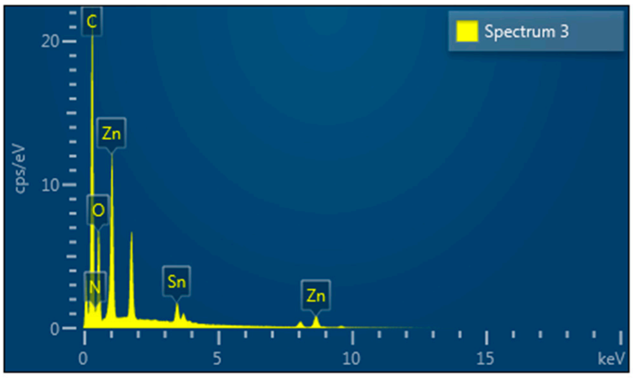

Element	Weight %	Atomic %
C	43.62	64.17
N	1.92	2.11
O	23.17	25.50
Zn	29.21	7.91
Sn	2.08	0.31
Total	100.00	100.00

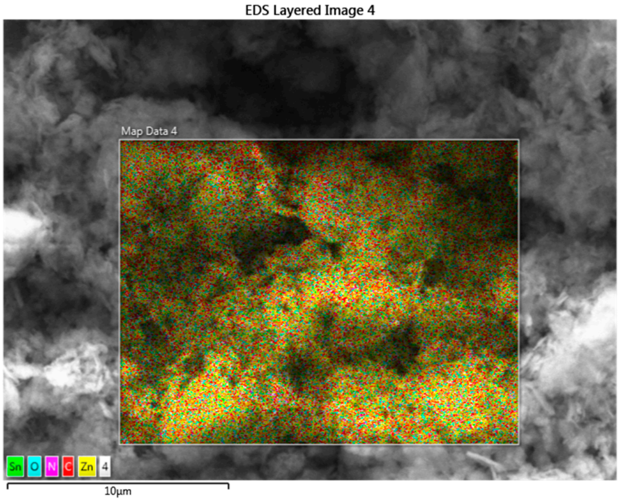

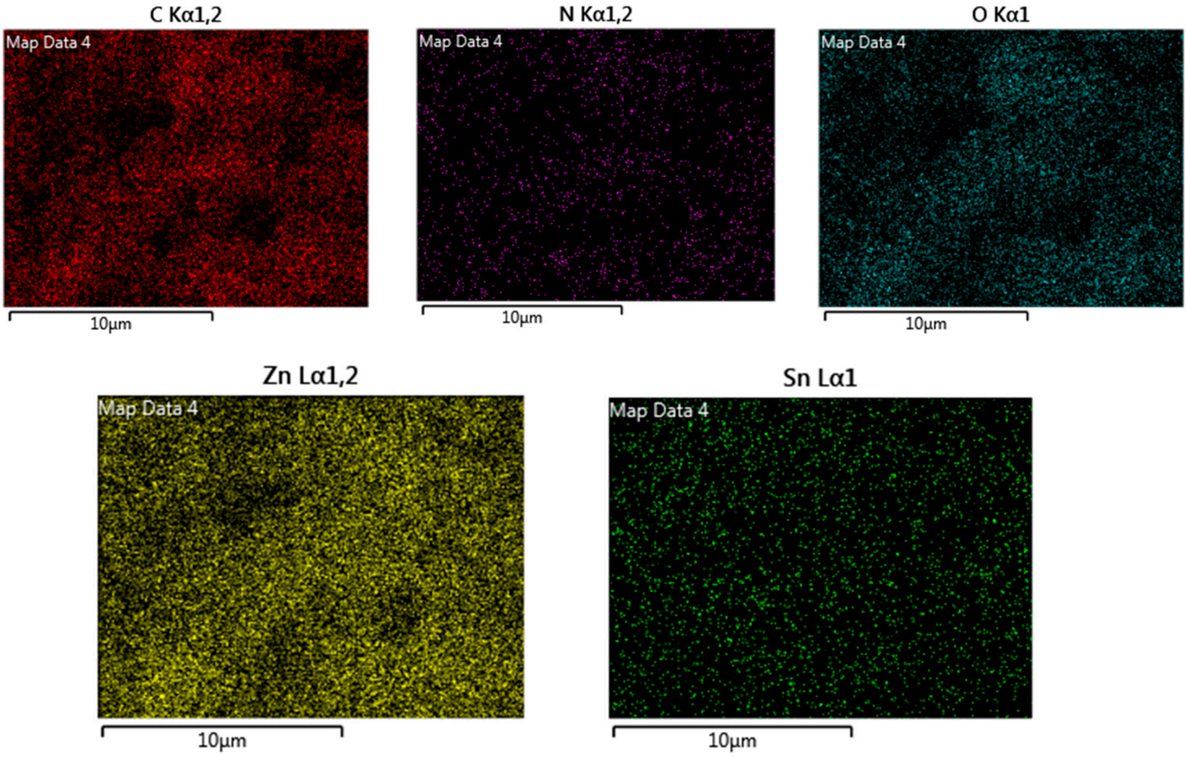

Figure S3. Energy dispersive X-ray spectroscopy (EDS) elemental maps (C, N, O, Zn, and Sn) of SnP/AA@ZnO.

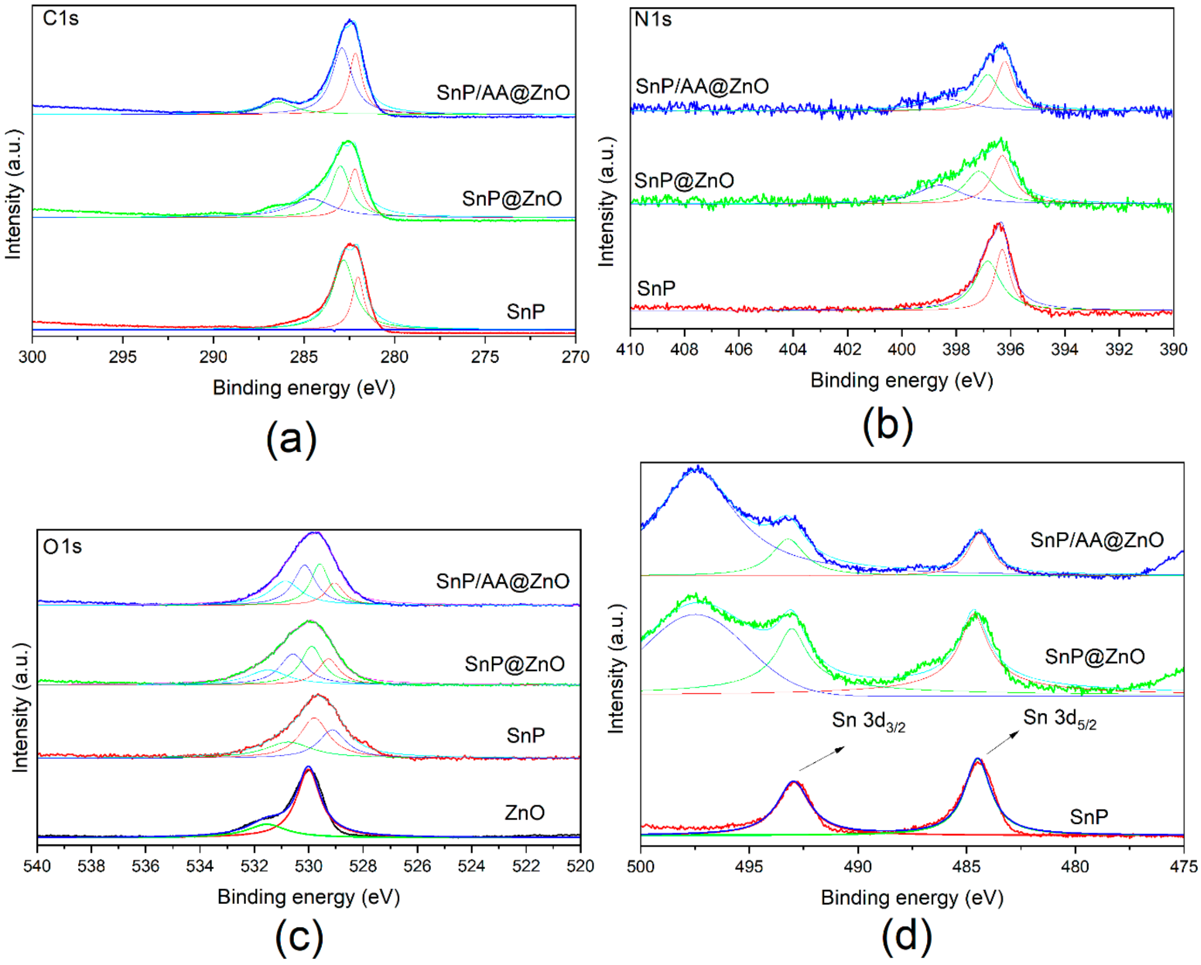

Figure S4. XPS spectra of SnP, ZnO, SnP@ZnO, and SnP/AA@ZnO. Deconvoluted profiles of (a) C 1s, (b) N 1s, (c) O 1s, (d) Sn 3d.

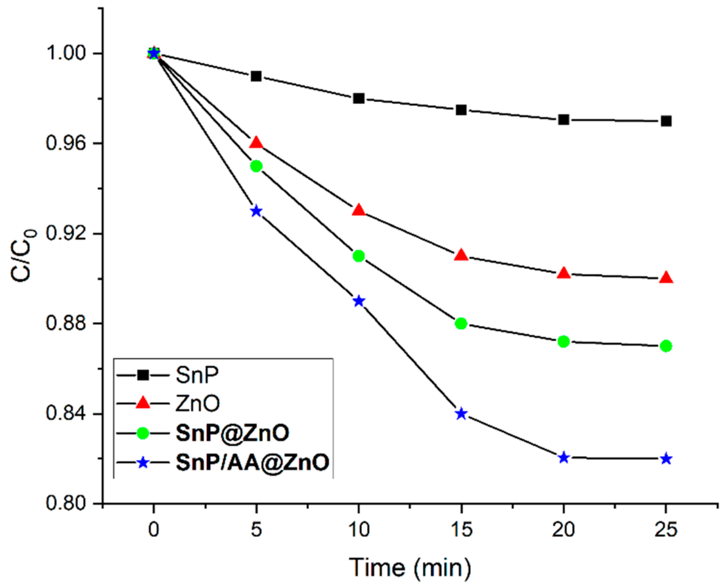

Figure S5. AM adsorption abilities of SnP, ZnO, SnP@ZnO, and SnP/AA@ZnO.

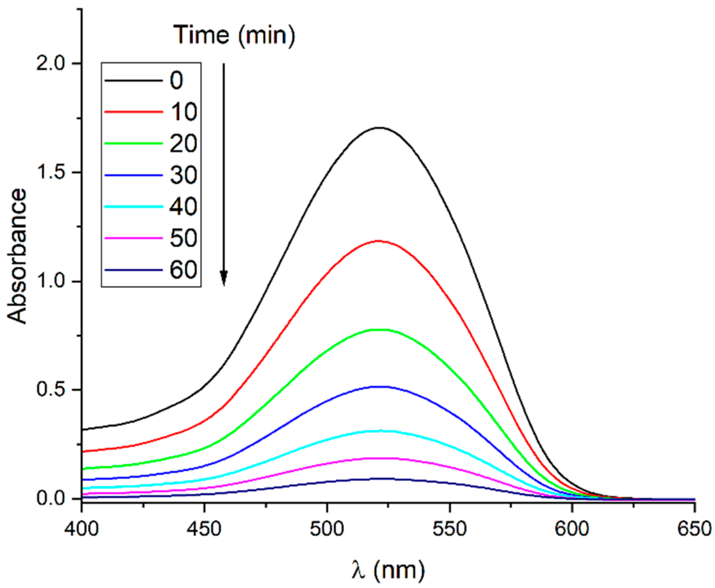

Figure S6. Photocatalytic degradation of AM dye in an aqueous solution by SnP/AA@ZnO under a visible light irradiation.

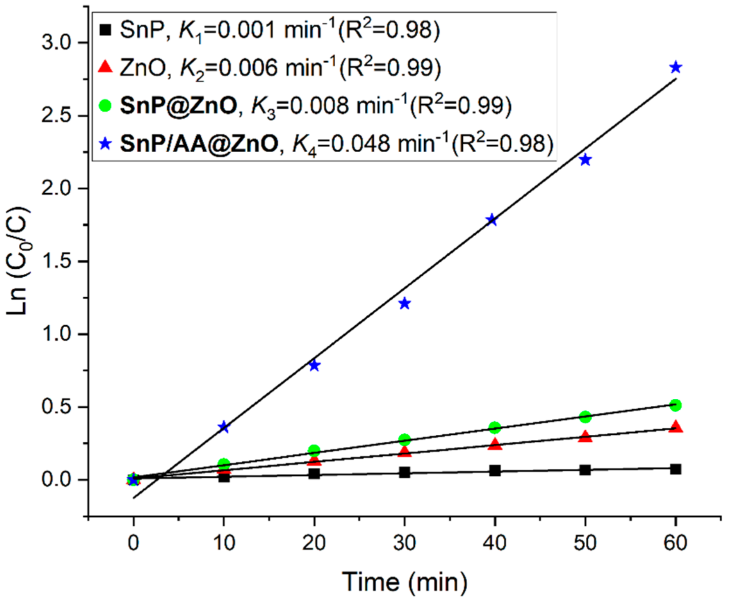

Figure S7. Kinetics for the photocatalytic degradation of AM dye by SnP, ZnO, SnP@ZnO, and SnP/AA@ZnO under a visible light irradiation.

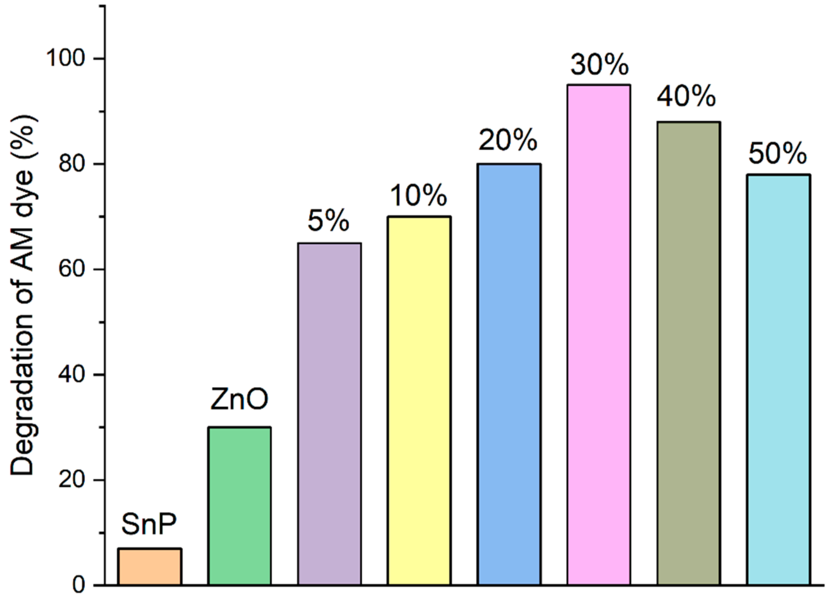

Figure S8. Comparison of AM dye degradations in the presence of SnP, ZnO, and SnP/AA@ZnO with various weight percentages of SnP with respect to ZnO.

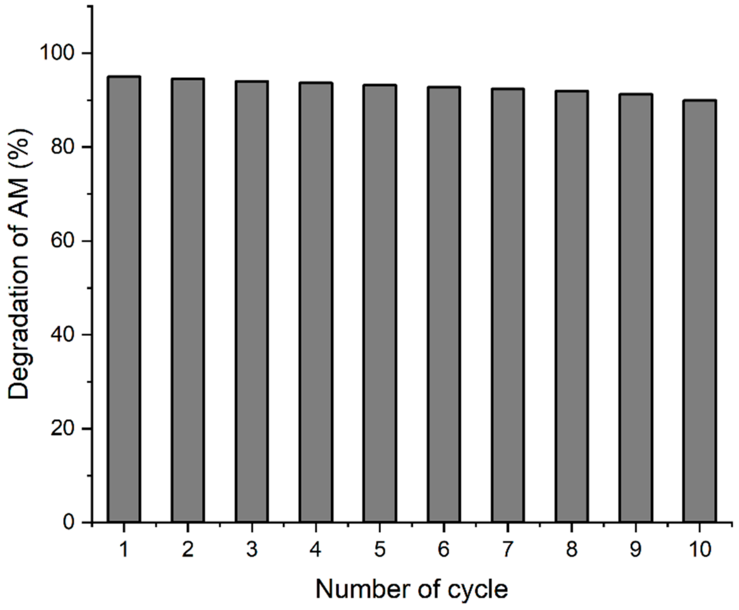

Figure S9. Recyclability of SnP/AA@ZnO in the degradation of AM dye.

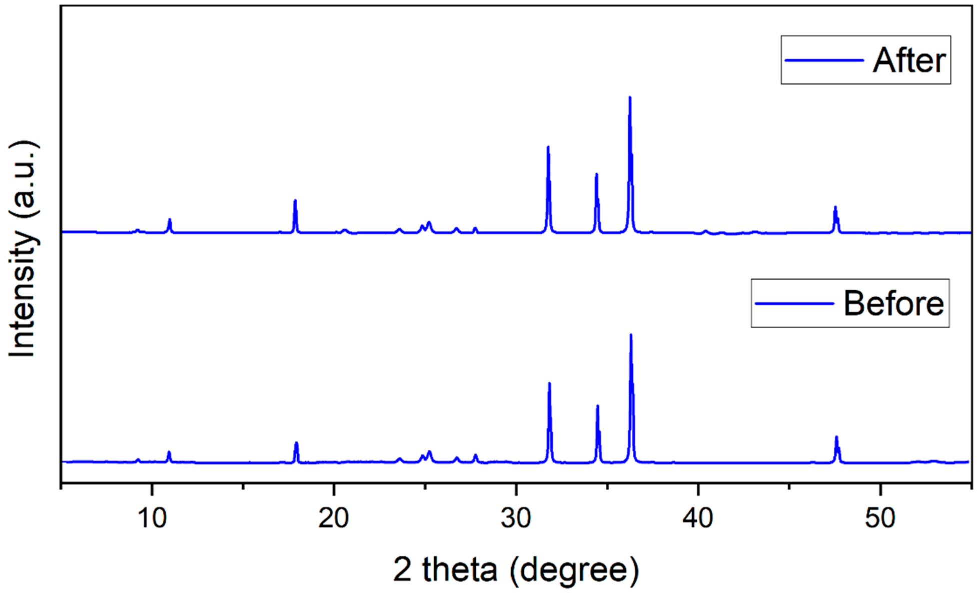

Figure S10. Powder XRD patterns of SnP/AA@ZnO after and before the AM photodegradation.

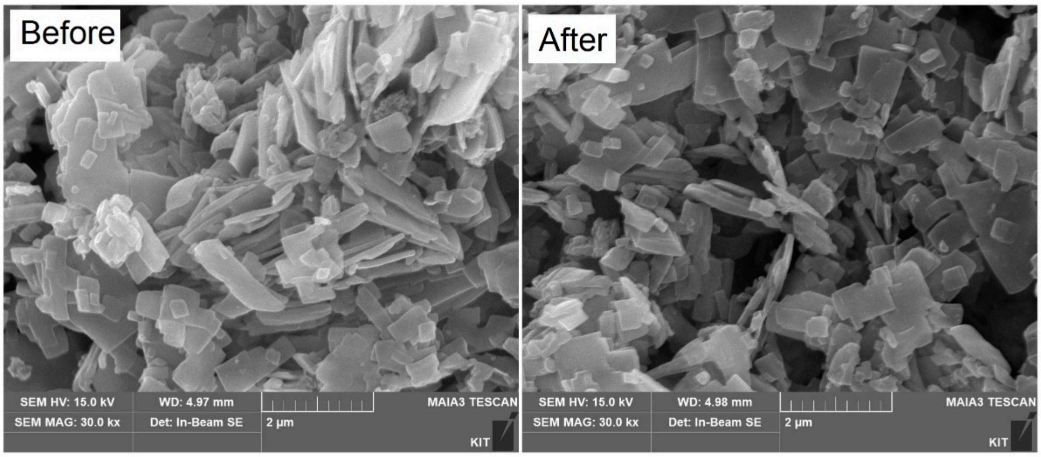

Figure S11. FE-SEM images of SnP/AA@ZnO before and after the degradation of AM dye.

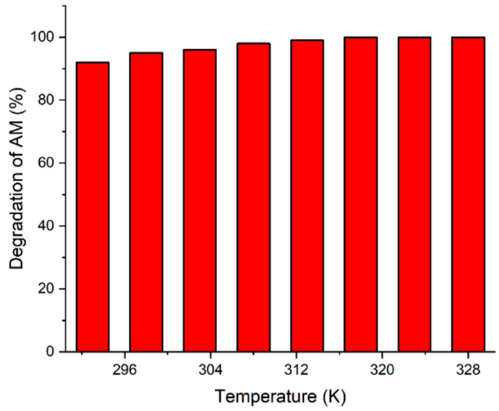

Figure S12. Effect of the temperature on the AM degradation in the presence of SnP/AA@ZnO.

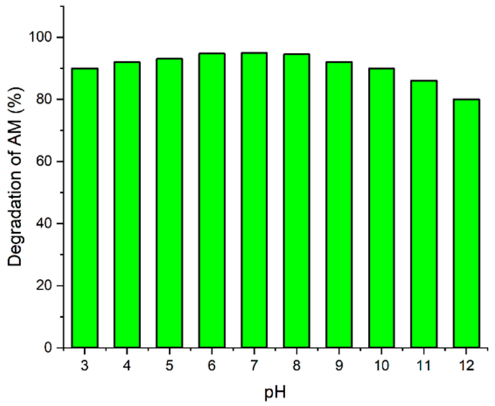

Figure S13. Effect of the pH of the solution on the degradation of AM dye in the presence of SnP/AA@ZnO.

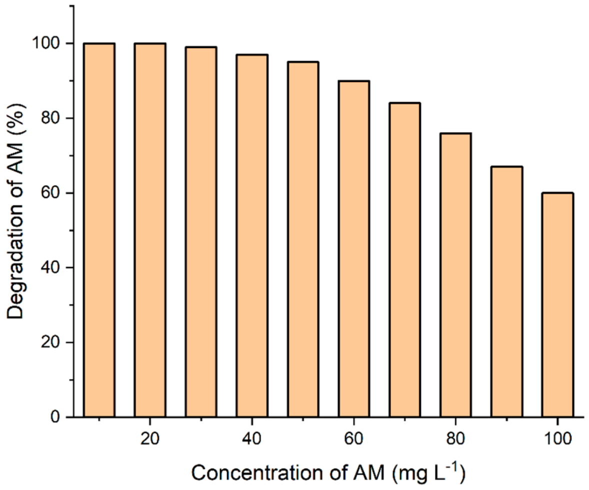

Figure S14. Effect of the initial concentration of the AM dye on the degradation with 50 mg of SnP/AA@ZnO.

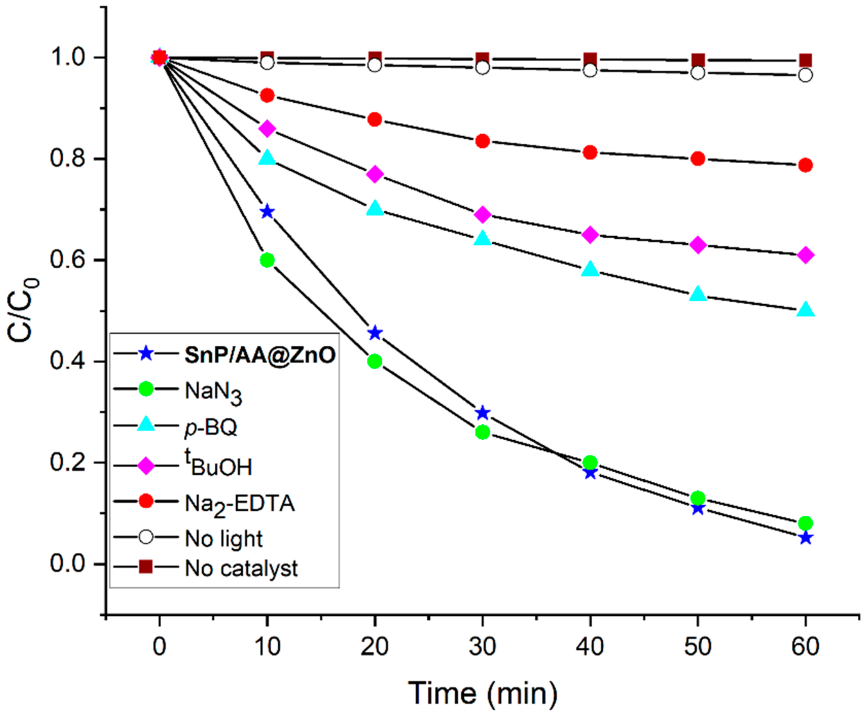

Figure S15. Photocatalytic degradation of AM dye in an aqueous solution by SnP/AA@ZnO with the addition of different scavengers under a visible light irradiation ([Na_2-EDTA]$_0$ = [*p*-BQ]$_0$ = [NaN_3]$_0$ = [^{t}BuOH]$_0$ = 1mM, pH = 7.0, T = 298 K).

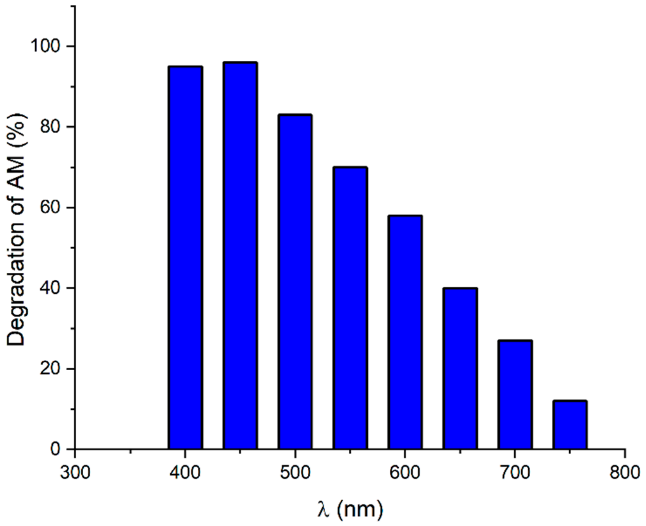

Figure S16. Photocatalytic activity of SnP/AA@ZnO at different wavelengths for the degradation of AM dye.

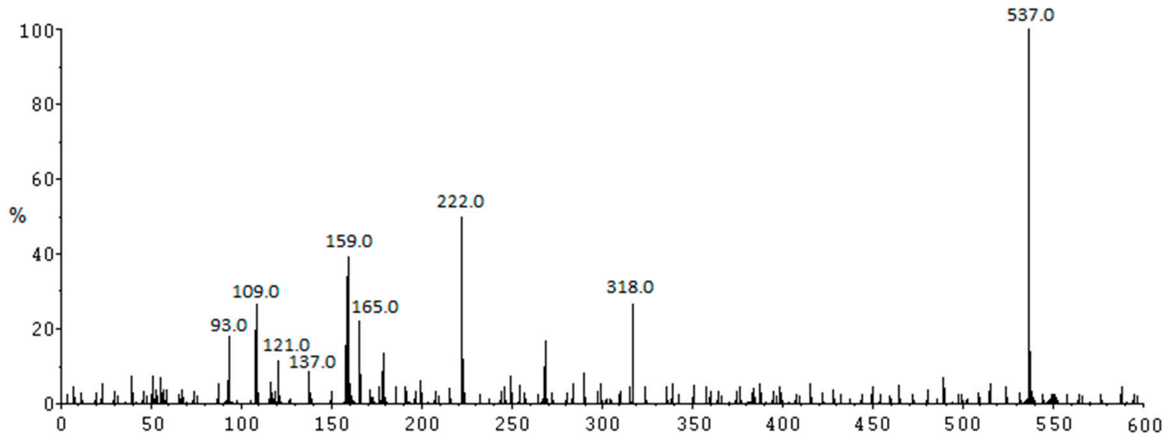

Figure S17. ESI-mass spectrum (negative ion mode) of the reaction mixture of AM dye with SnP/AA@ZnO after 30 min of visible light irradiation.
